# Chronic pain in mental disorders: An umbrella review of the prevalence, risk factors, and treatments across 957,168 people with mental disorders and 16,606,910 controls

**DOI:** 10.1192/j.eurpsy.2025.10074

**Published:** 2025-08-12

**Authors:** Brendon Stubbs, Ruimin Ma, Marco Solmi, Nicola Veronese, Tine Van Damme, Eugenia Romano, Robert Stewart, Nilufar Mossaheb, José Francisco López-Gil, Joseph Firth, Davy Vancampfort

**Affiliations:** 1 Department of Psychological Medicine, Institute of Psychiatry, Psychology and Neuroscience, King’s College London, London, UK; 2 Center for Sport Science and University Sports, University of Vienna, Vienna, Austria; 3 SCIENCES Lab, Department of Psychiatry, University of Ottawa, Ottawa, ON, Canada; 4 Regional Centre for the Treatment of Eating Disorders and On Track: The Champlain First Episode Psychosis Program, Department of Mental Health, The Ottawa Hospital, ON, Canada; 5 Ottawa Hospital Research Institute (OHRI) Clinical Epidemiology Program, University of Ottawa, ON, Canada; 6 Department of Child and Adolescent Psychiatry, Charité Universitätsmedizin, Berlin, Germany; 7 Saint Camillus International, University of Health Sciences, Rome, Italy; 8 Department of Internal Medicine, Geriatrics Section, University of Palermo, Palermo, Italy; 9 Department of Rehabilitation Sciences, KU Leuven, Leuven, Belgium; 10 University Psychiatric Center KU Leuven, Leuven, Belgium; 11 School of Medicine, Universidad Espíritu Santo, Samborondón, Ecuador; 12 Vicerrectoría de Investigación y Postgrado, Universidad de Los Lagos, Osorno, Chile; 13 Division of Psychology and Mental Health, University of Manchester, Manchester Academic Health Science Centre, Manchester, UK; 14 Greater Manchester Mental Health NHS Foundation Trust, Manchester, UK; 15 Leuven Brain Institute, Leuven, Belgium

**Keywords:** Chronic pain, mental illness, anxiety, depression, psychosis

## Abstract

**Background:**

Chronic pain (CP) and mental disorders often coexist, yet their relationship lacks comprehensive synthesis. This first hierarchical umbrella review examined systematic reviews and meta-analyses, also observational studies and randomized controlled trials (where reviews are currently lacking) to report CP prevalence, risk factors, and treatment across mental disorders.

**Methods:**

We searched MEDLINE, PsycINFO, Embase, Web of Science, and CINAHL, identifying 20 studies on anxiety, depression, bipolar disorder, schizophrenia, ADHD, autism, or dementia, and CP. Quality was assessed using AMSTAR and Newcastle-Ottawa Scale.

**Results:**

Prevalence varied widely—23.7% (95% CI 13.1–36.3) in bipolar disorder to 96% in PTSD—consistently exceeding general population rates (20–25%). Risks were elevated, with bidirectional links in depression (OR = 1.26–1.88). Risk factors included female gender, symptom severity, and socioeconomic disadvantage, though data were limited beyond PTSD and depression. Treatment evidence was sparse: cognitive behavioral therapy showed small effects on pain (SMD = 0.27, 95% CI -0.08–0.61), acupuncture with medication improved pain (MD = -1.06, 95% CI -1.65–-0.47), and transcranial direct current stimulation reduced pain in dementia (d = 0.69–1.12). Methodological issues were evident, including heterogeneous designs and inconsistent pain definitions.

**Conclusions:**

This review confirms CP as a significant comorbidity in mental disorders. Clinicians should prioritize routine pain screening and multimodal treatments. Researchers need longitudinal studies with standardized assessments to clarify causality and improve interventions. Taken together, this work highlights an urgent need for integrated psychiatric care approaches, emphasizing that addressing CP could enhance mental health outcomes and overall patient well-being.

## Introduction

Chronic pain, defined as an unpleasant sensory and emotional experience associated with actual or potential tissue damage that persists or recurs for more than three months [1], is a pervasive global health challenge. Affecting approximately 25% of the world’s population [2], it ranks among the leading causes of disability, with conditions such as osteoarthritis, chronic low back pain, and headaches consistently driving medical consultations [3]. The societal and economic burden is immense, with annual costs in the United States alone estimated at US$560–635 billion due to medical expenses and lost productivity [4]. Beyond its direct impact, chronic pain disproportionately affects certain populations, including women, individuals from lower socioeconomic backgrounds, veterans, and rural communities, exacerbating existing health inequities [5].

Once regarded primarily as a symptom, chronic pain is now recognized as a distinct disease state, driven by overlapping mechanisms, that is nociceptive pain resulting from tissue injury, neuropathic pain arising from nerve damage, and nociplastic pain stemming from nervous system sensitization [6]. This paradigm shift underscores the need for tailored, multidimensional treatment strategies. The complexity of chronic pain is further compounded by its bidirectional relationship with mental disorders, a well-documented yet insufficiently addressed issue in clinical practice [7]. There is some tentative evidence suggesting that individuals with conditions such as depression, anxiety, schizophrenia, bipolar disorder (BD), autism, attention-deficit/hyperactivity disorder (ADHD), and dementia may experience a higher prevalence of chronic pain (35–50%) compared to the general population [8, 9]. This comorbidity operates through multiple pathways. Chronic pain exacerbates psychological distress, while mental disorders heighten pain perception and reduce pain tolerance, creating a vicious cycle that complicates both diagnosis and management [10]. The consequences of this interplay are severe. Individuals with comorbid chronic pain and mental disorders face worse clinical outcomes, greater healthcare utilization, and higher economic burden than those with either condition alone [11]. A 2020 analysis found that chronic pain in individuals with depression and anxiety incurs an additional annual cost of $5,208 per patient, exceeding the financial burden associated with cardiovascular disease, diabetes, or obesity, primarily due to increased emergency visits and productivity losses [12]. Pain sensitivity also varies across mental disorders, further complicating care. Individuals with schizophrenia, for example, often exhibit elevated pain thresholds and tolerance, which may delay the diagnosis of serious conditions such as cancer [13]. Conversely, those with depression or anxiety typically display heightened pain sensitivity, intensifying their suffering [14].

Despite these insights, significant gaps remain in understanding the prevalence, risk factors, and optimal treatment of chronic pain in psychiatric populations. Diagnostic overshadowing, in which physical pain is misattributed to mental illness, frequently delays intervention [15], while the analgesic potential of psychiatric medications such as antidepressants and antipsychotics remains underexplored in this context [16]. Addressing these deficiencies requires a comprehensive evaluation of the relationship between chronic pain and mental disorders, yet existing evidence is fragmented across multiple sources. To bridge this gap, this hierarchical umbrella review synthesizes high-quality findings from systematic reviews and meta-analyses, as well as large cohort studies and randomized controlled trials (RCTs) where reviews are unavailable. It aims to examine disorder-specific patterns and gaps in the prevalence and risk factors and treatments effectiveness of chronic pain across diverse mental disorders, in order to inform integrated care approaches. By consolidating top-tier evidence, this study aims to advance clinical practice and policy, promoting a more biopsychosocial approach to managing this complex comorbidity.

## Methods

### Overview

We conducted a hierarchical umbrella review to synthesize evidence from systematic reviews and meta-analyses or large observation or trial evidence on the prevalence, risk factors, and treatment outcomes of chronic pain in adults with mental disorders (International Prospective Register of Systematic Reviews [PROSPERO] CRD420251026088). This approach enabled a comprehensive assessment of the top-tier evidence, focusing on anxiety disorders, depression, BD, schizophrenia, autism, ADHD, personality disorders, and other mental disorders.

### Search strategy

Four reviewers (BS, RM, ER, and DV) independently searched Medical Literature Analysis and Retrieval System Online (MEDLINE) (via PubMed), PsycINFO, Embase, Web of Science, and Cumulative Index to Nursing and Allied Health Literature (CINAHL) for studies published between January 2000 and 1 February 2025. Search terms combined mental disorder-specific keywords (e.g., “mental illness,” “schizophrenia,” “depression,” “bipolar disorder,” “ADHD,” “personality disorder”) with pain-related terms (e.g., “chronic pain,” “persistent pain,” “musculoskeletal pain”) using Boolean operators. A sample search string for MEDLINE was: (“mental disorders” OR “schizophrenia” OR “depression” OR “bipolar disorder” OR “ADHD”) AND (“chronic pain” OR “persistent pain”) AND (systematic review OR meta-analysis). No language restrictions were applied, though only English-language publications were ultimately included due to resource constraints. Reference lists of included studies were hand-searched to identify additional relevant reviews.

### Inclusion and exclusion criteria

Eligible studies were systematic reviews and meta-analyses published between 2010 and 2025, focusing on adults (≥18 years) with mental disorders diagnosed per standardized criteria (Diagnostic and Statistical Manual of Mental Disorders [DSM]-IV, DSM-5, International Classification of Diseases [ICD]-10, or ICD-11). Chronic pain was defined as pain persisting or recurring for more than three months, encompassing all pain types and locations [17]. Where systematic reviews were unavailable, large-scale observational studies or RCTs were considered. Studies were required to report on at least one primary outcome: prevalence of chronic pain, risk compared to the general population or other psychiatric conditions, risk factors, or treatment effectiveness. Secondary outcomes included quality of life, functional outcomes, healthcare utilization, and economic burden. Narrative reviews, case studies, and studies lacking a systematic methodology were excluded. In order to meet the criteria for chronic pain, only studies that reported pain symptoms for at least three months were included or a specific clinical diagnosis of chronic pain.

### Study selection

Two independent reviewers (RM and ER) screened titles and abstracts, followed by a full-text review of potentially eligible studies. Discrepancies were resolved by a third reviewer.

### Data extraction

Data were extracted by two independent reviewers (RM and ER) using a standardized form capturing study characteristics (e.g., authors, year, design), population details (e.g., mental disorder type, diagnostic criteria), pain definitions and assessment methods, and outcomes (e.g., prevalence, risk ratios, intervention effects). Effect sizes, confidence intervals (CIs), and heterogeneity measures (e.g., I^2^) were recorded where reported. Extraction was performed independently by two reviewers, with inconsistencies reconciled through discussion. A third author was available for discussion if needed.

### Quality assessment

Two independent reviewers (RM and ER) assessed the methodological quality of included systematic reviews using A MeaSurement Tool to Assess systematic Reviews (AMSTAR) tool, which is a validated 11-item checklist developed to appraise the quality of systematic reviews and meta-analyses, and it has been widely used for reviews in healthcare interventions [18]. Additionally, we applied the AMSTAR+ framework as a secondary grading system to account for complex methodological characteristics of the included reviews. All included reviews were rated as high, moderate, low, or critically low quality based on the AMSTAR and AMSTAR+ criteria. These assessments were used to *contextualize the strength and reliability of findings during synthesis and interpretation.* For primary studies included in the absence of reviews, risk of bias was assessed using the Newcastle Ottowa Scale (NOS) [19] for observational studies and Physiotherapy Evidence Database (PEDro) [20] for randomized trials.

### Data synthesis

Findings were synthesized narratively by mental disorder category, supported by tables summarizing prevalence, risk factors, and treatment outcomes. Subgroup analyses explored variations by mental disorder subtype, pain type/location, treatment modality, study quality, and geographical region. Evidence consistency and strength were evaluated to contextualize findings.

## Results

### Search results

**Figure 1.**
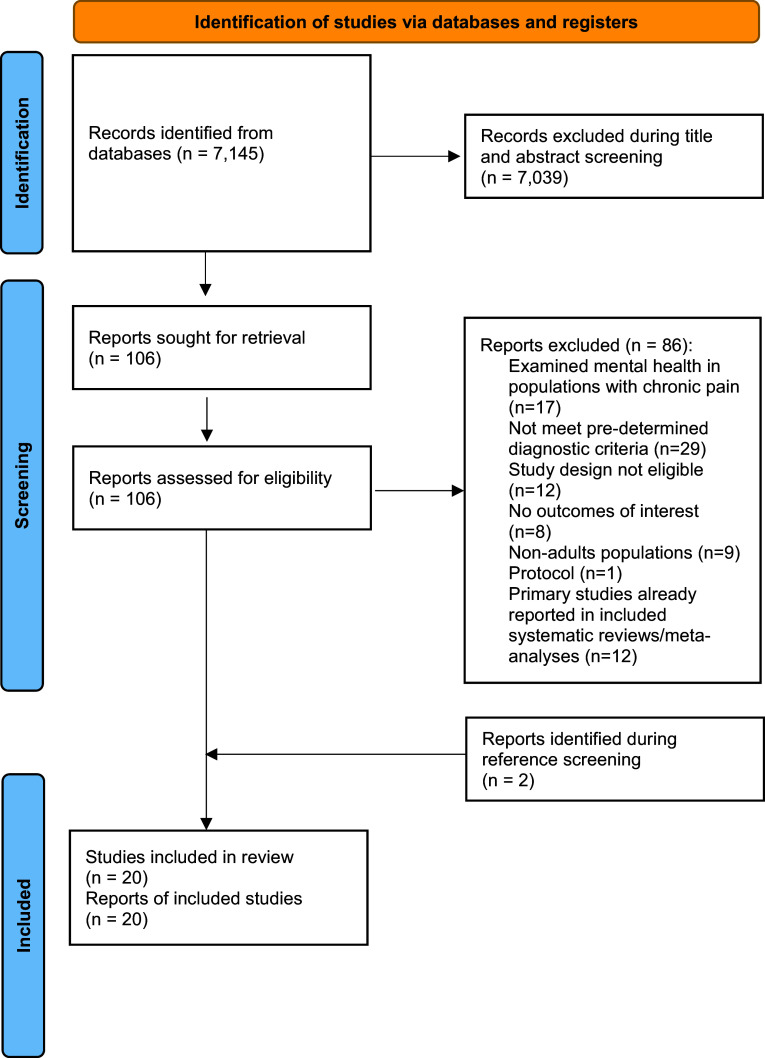
PRISMA 2020 flow diagram for new systematic reviews which included searches of databases and registers only. *Consider, if feasible to do so, reporting the number of records identified from each database or register searched (rather than the total number across all databases/registers). **If automation tools were used, indicate how many records were excluded by a human and how many were excluded by automation tools. *From:* Page MJ, McKenzie JE, Bossuyt PM, Boutron I, Hoffmann TC, Mulrow CD, et al. The PRISMA 2020 statement: an updated guideline for reporting systematic reviews. *BMJ* 2021;372:n71. doi: 10.1136/bmj.n71 For more information, visit: http://www.prisma-statement.org/

The total number of eligible hits was 7,145, of these, 106 full-text articles were screened. Main reasons for exclusion were examining mental disorders in populations with chronic pain (*n* = 17), mental disorders or chronic pain fail to meet pre-determined diagnostic criteria (*n* = 29), study design not eligible (*n* = 12), no outcomes of interest (*n* = 8), studies focused on non-adults populations (*n* = 9), protocol (*n* = 1) and primary studies that were reported in included systematic reviews/meta-analyses (*n* = 12). Two additional studies were retrieved from reference list manual searching, resulting in a final number of 20 studies being included in this umbrella review. The study selection process is illustrated in the Preferred Reporting Items for Systematic reviews and Meta-Analyses (PRISMA) flow diagram (Figure 1). Sample sizes ranged from a small cohort (*n* = 40 [21]) to large meta-analysis of population-based studies (*n* = 12,375,644 [22]). Eight systematic reviews [22–29] scored 7–10/11 on AMSTAR, indicating moderate to high quality, though AMSTAR+ scores (where available) ranged from 1–3/7 due to limitations in double-blinding, sample size, and heterogeneity. One RCT [21] achieved an excellent PEDro score of 10/10, while cohort studies scored 2–7/9 on NOS, with strengths in representativeness but weaknesses in comparability and follow-up.

### Anxiety and stress disorders

There were three studies on anxiety and stress disorders [24, 27, 30], including two on prevalence [24, 30], one on risk factors [24], and two on treatments [24, 27], totaling 22,335 participants (*n* posttraumatic stress disorder (PTSD) = 11,200; *n* controls = 11,135).

#### Prevalence of chronic pain and versus controls

Two studies explored chronic pain prevalence in anxiety and stress disorders, primarily PTSD, ranging from 66 to 96%. Shipherd et al. [30], in an observational study (*k* = 1, *n* = 85, NOS = 5/9), reported 66% (*n* = 56/85) of PTSD patients had chronic pain, with chronic low back pain (19%) and osteoarthritis (9%) prevalent. Rometsch-Ogioun El Sount et al. [24], in a systematic review and meta-analysis (SR & MA) (*k* = 15, *n* PTSD = 10,931 versus *n* controls = 11,135, AMSTAR = 8/11, AMSTAR+ = 1/7) found a higher prevalence of 88–96%, with common sites including stomach pain (87.7%), chest pain (84.4%), and back pain (82.2%), significantly elevated compared to controls.

#### Risk factors for chronic pain

One study addressed risk factors. Rometsch-Ogioun El Sount et al. [24] identified older age (*β* = 0.206, *p* = 0.008), female gender (*β* = 0.206, *p* = 0.008), socioeconomic difficulties (*β* = 0.159, *p* = 0.047), and severe PTSD symptoms (*β* = 0.217–0.277, *p* = 0.028–0.005) as significant predictors.

#### Treatment options

Two studies examined treatments. Rometsch-Ogioun El Sount et al. [24] found 6 intervention studies and reported an RCT where six sessions of Emotional Freedom Technique reduced pain scores from 4.78/10 to 2.94/10 after six months (*p* < 0.001). Ma et al. [27], in an SR (*k* = 26, AMSTAR = 10/11), found that among four PTSD trials (*k* = 4, *n* PTSD =184), one body-based intervention, likely involving physical activities such as yoga or tai chi, demonstrated a very large effect in reducing pain severity. Additionally, one pharmacological therapy, possibly utilizing antidepressants or anti-seizure medications, improved pain severity. Both interventions also provided significant benefits in reducing pain interference and disability, enhancing daily functioning, and minimizing the impact of pain on physical activities.

### Major depressive disorder

There were eight studies in depression [25–27, 31–35], including five on prevalence [31–35], four on risk factors [32–35], and three on treatments [25–27], totaling 493,281 participants (*n* major depressive disorder (MDD) = 376,872, *n* control = 116,409).

#### Prevalence of chronic pain and versus controls

Five studies assessed chronic pain prevalence in MDD, ranging from 53.8 to 65%. Hanssen et al. [31], in an observational study (*k* = 1, *n* MDD = 275, NOS = 4/9), found 53.8% (*n* = 148/275) among older adults, with joint pain (37.8%) and back pain (28.4%) common. Mendelian randomization studies by Zhu et al. [32] (*k* = 1, *n* = 341,797, NOS = 4/9) and Li et al. [33] (*k* = 1, *n* = 225 SNPs for multisite pain, *n* = 69 SNPs for MDD, NOS = 4/9) showed MDD increased chronic regional pain risk (OR = 1.26, 95% CI 1.16–1.38) and multisite pain raised MDD risk (odds ratio [OR] = 1.88, 95% CI 1.64–2.15) versus controls. Nicholl et al. [34] (*k* = 1, *n* MDD = 31,814, *n* control = 116,184, NOS = 3/9) noted 24.3% (*n* = 7,736) with single-site pain and 1.9% (*n* = 597) with widespread pain. Rambla et al. [35], in a secondary RCT analysis (*k* = 1, *n* = 317, NOS = 7/9), contributed prevalence data implicitly through pain severity measures.

#### Risk factors for chronic pain

Four studies identified risk factors. Nicholl et al. [34] observed higher pain comorbidity in White participants (11.1%) than Black (8.5%) or Asian (7.5%), with a stronger association in Black individuals (OR = 1.86, 95% CI 1.52–2.27). Zhu et al. [32] linked insomnia (effect size = 1.04) to risk. Rambla et al. [35], in a secondary RCT analysis highlighted baseline pain severity (*β* = 0.53, 95% CI 0.37–0.68), prolonged pain (>2 years, *β* = 0.91, 95% CI 0.11–1.71), depression severity (*β* = 0.58, 95% CI 0.04–1.11), and pain catastrophizing (*β* = 0.03, 95% CI 0.00–0.05) as predictors, with employment protective (*β* = −0.77, 95% CI -1.52–0.02). Li et al. [33] associated sedentary behaviour (TV watching: OR = 1.46, 95% CI 1.39–1.53) with increased risk.

#### Treatment options

Three studies evaluated treatments. Patel and Chrisinger [25], in an SR & MA (*k* = 7, *n* MDD = 891, AMSTAR = 9/11, AMSTAR+ = 1/7), found psychosocial interventions (e.g., cognitive behavioural therapy [CBT] had limited pain impact (standardised mean difference [SMD] = 0.27, 95% CI−-0.08 to 0.61, i.e., small effect). You et al. [26] (*k* = 8, *n* MDD = 636, AMSTAR = 10/11, AMSTAR+ = 2/7) showed acupuncture plus drugs outperformed single-drug therapy for depression (mean difference [MD] = -2.95, 95% CI −3.55 to −2.36) and pain (MD = −1.06, 95% CI −1.65 to -0.47). Ma et al. [27] reported that among 11 depression trials (*k* = 11, *n* MDD = 1,073), CBT-based interventions demonstrated small to medium effects on pain severity (SMD typically 0.2–0.5), while body-based interventions, such as yoga or tai chi, showed large effects (SMD > 0.8). Pharmacological interventions, involving antidepressants or anti-seizure medications, reduced pain severity in two out of three trials. These treatments also improved functional outcomes, reducing pain interference with daily activities and decreasing pain catastrophizing.

### Comorbid depression and anxiety

There were two studies in comorbid depression and anxiety [23, 27], including one on prevalence [23] and one on treatments [27], totaling 15,212 participants (*n* depression & anxiety = 7,549; *n* controls = 7,663).

#### Prevalence of chronic pain and versus controls

One study investigated this, with prevalence ranging from 50 to 60%. Brandl et al. [23], in an SR & MA (*k* = 1, *n* depression & anxiety = 7,517, *n* controls = 7,663, AMSTAR = 8/11, AMSTAR+ = 2/7), reported 50–60% comorbidity with chronic pain (e.g., low back pain, fibromyalgia), higher than controls.

#### Risk factors for chronic pain

No specific risk factors were detailed for this group.

#### Treatment options

One study addressed treatments. In a systematic review by Ma et al. [27], one trial (*k* = 1, *n* depression & anxiety = 32) on Mindfulness-Based Intervention (MBI) was highlighted. The MBI trial, involving mindfulness-based stress reduction (MBSR) or mindfulness-based cognitive therapy (MBCT), showed no significant reduction in pain severity but demonstrated a large decrease in pain interference. This suggests that while the intensity of pain remained similar, the intervention substantially reduced the impact of pain on daily activities and quality of life, offering a functional benefit for patients.

### Bipolar disorder

There were two studies in BD [22, 36], including one on prevalence [22], one on risk factors [36], totaling 12,405,894 participants (*n* BD = 201,602; *n* controls = 12,204,292).

#### Prevalence of chronic pain and versus controls

One study examined chronic pain in BD, with a prevalence of 23.7%. Stubbs et al. [22], in a meta-analysis (*k* = 22, *n* BD = 171,352, *n* controls = 12,204,292, AMSTAR = 10/11, AMSTAR+ = 3/7), reported 23.7% (95% CI 13.1–36.3%) and a relative risk (RR) of 2.14 (95% CI 1.67–2.75) versus controls.

#### Risk factors for chronic pain

One study assessed risk factors. Trivedi et al. [36], in a cross-sectional study (*k* = 1, *n* BD + chronic pain = 15,125, *n* BD =15,125, NOS = 6/9), noted BD patients with chronic pain were older (47.6 versus 40.4 years), more often female (58.4% versus 55.2%), and White (77.2% versus 66.7%).

#### Treatment options

No specific treatment outcomes were reported for BD alone.

### Schizophrenia

There were two studies in schizophrenia [29, 37], both on prevalence, totaling 4,501,993 participants (*n* schizophrenia = 242,782; *n* controls = 4,259,211).

#### Prevalence of chronic pain and versus controls

Two studies assessed chronic pain in schizophrenia, with prevalence ranging from 29.5 to 33.3%. Stubbs et al. [29], in a meta-analysis (*k* = 14, *n* schizophrenia = 242,703, *n* controls = 4,259,221, AMSTAR = 10/11), reported individual prevalence rates from 1.8 to 80%. The meta-analysis also reported a pooled prevalence of 29.5% (95% CI 7.4–51.6%) across four studies [38–41], and an RR of 1.65 (95% CI 0.68–3.99) versus controls based on two studies. Sepulveda-Torres et al. [37], in a cross-sectional study (*k* = 1, *n* = 79, NOS = 2/9), found head/face/mouth pain (33.3%) prevalent, though limited by sample size.

#### Risk factors for chronic pain

No specific risk factors were identified for schizophrenia.

#### Treatment options

No targeted treatment outcomes were reported for schizophrenia alone.

### Attention-deficit/hyperactivity disorder

There were two studies on ADHD [28, 42], both on prevalence [28, 42] and one on risk factors [42], totaling 8,463 participants (*n* ADHD = 263, *n* controls = 8,200).

#### Prevalence of chronic pain and versus controls

Two studies investigated chronic pain in ADHD, with prevalence ranging from 29.1 to 66.9%. Battison et al. [28], in a scoping review (*k* = 11, *n* not fully specified, AMSTAR = 7/11), reported 29.1–66.9%, often musculoskeletal or multisite pain. Mundal et al. [42], in a 9-year longitudinal study (*k* = 1, *n* ADHD = 263, *n* controls = 8,200, NOS = 3/9), found 66.5% (*n* = 175/263) versus 44.5% in controls.

#### Risk factors for chronic pain

One study addressed risk factors. Mundal et al. [42] (*k* = 1, *n* ADHD = 263, *n* controls = 8,200, NOS = 3/9) highlighted female sex as a risk factor.

#### Treatment options

No specific treatment results were available for ADHD alone.

### Attention-deficit/hyperactivity disorder and/or autism

There was one study on ADHD and/or autism [43], covering prevalence and risk factors, with 77 participants in total.

#### Prevalence of chronic pain and versus controls

One study explored this, reporting a prevalence of 76.6%. Asztély et al. [43], in an observational study (*k* = 1, *n* = 77, NOS = 5/9), found 76.6% (*n* = 59/77) in a mixed ADHD/autism cohort, with chronic widespread pain at 32.5% and lower back pain (46.8%) most common; no control group was included.

#### Risk factors for chronic pain

One study assessed risk factors. Asztély et al. [43] noted no significant differences in abdominal pain or headaches between ADHD and autism, but stimulant use (32.4% of ADHD patients) reduced chronic widespread pain (16.7% versus 42%).

#### Treatment options

No specific treatments were detailed beyond the stimulant effect.

### Dementia

There was one study on dementia [44], focused on treatments, with 40 participants in total.

#### Prevalence of chronic pain and versus controls

A single study assessed chronic pain in dementia, though prevalence was not quantified. Martorella et al. [44], in an RCT (*k* = 1, *n* = 40, PEDro = 10/10), evaluated pain in early-stage dementia without specific rates.

#### Risk factors for chronic pain

No risk factors were specified.

#### Treatment options

One study examined treatments. Martorella et al. [44] (*n* active tDCS = 20, *n* sham = 20) found transcranial direct current stimulation (tDCS) significantly reduced pain intensity (Cohen’s *d* = 0.69 on NRS, i.e., moderate effect; Cohen’s *d* = 1.12 on Mobilization-Observation-Behavior-Intensity-Dementia, i.e., large effect).

### Severe mental illness

There was one study on severe mental illness (SMI) [45], focusing on prevalence and treatment, totaling 116,783 participants (*n* MDD = 65,750; *n* BD = 38,117; *n* schizophrenia = 12,916).

#### Prevalence of chronic pain and versus controls

Owen-Smith et al. [45], in a longitudinal study using medical records (*k* = 1, *n* = 116,783, NOS = 5/9), reported chronic pain prevalence across SMI: 62.4% (*n* = 41,036/65,750) in MDD, 61.5% (*n* = 23,423/38,117) in BD, and 47.2% (*n* = 6,092/12,916) in schizophrenia. Common pain types included limb/extremity arthritis (32.3% MDD, 31.6% BD, 22.8% schizophrenia), back pain (20.4% MDD and BD, 14.4% schizophrenia), and abdominal/bowel pain (15.1% MDD, 15.2% BD, 11.6% schizophrenia), with no control group comparison provided.

#### Risk factors for chronic pain

No specific risk factors were identified for SMI in this study.

#### Treatment options

Owen-Smith et al. [45] reported opioid dispensing rates alongside prevalence: 36.9% of MDD patients, 45.5% of BD patients, and 27.2% of schizophrenia patients with chronic pain received opioids, indicating a reliance on pharmacological management, though specific efficacy outcomes were not detailed.

A summary of chronic pain characteristics, risk factors, and treatment options across each mental disorder are presented in Table 1. The quality assessment of the included studies is summarised in Tables 2–4. Please see Supplementary Tables S3–S5 for detailed characteristics and results of included reviews and studies reporting prevalence/risk, risk factors, and treatments of chronic pain across different mental illnesses, respectively.

**Table 1.** Synthesis of chronic pain prevalence, risk factors and treatments by mental disorders

Mental Disorder	Sample size	Pain characteristics	Primary risk factors	Treatment approaches	Treatment outcomes
**Anxiety and Stress Disorders (PTSD)**	Total n =22,335 N PTSD =11,200 N controls =11,135	Prevalence: 66^30^–96%^24^; Common Types: Stomach pain (87.7%)^24^, chest pain (84.4%)^24^, back pain (82.2%)^24^, arms/legs (85.0%)^24^, headaches (85.7%)^24^	Older age^24^, female gender^24^, socioeconomic difficulties^24^, severe PTSD symptoms^24^	Emotional Freedom Technique (EFT)^24^, body-based interventions^27^, pharmacological therapy^27^	EFT: Reduced pain scores from 4.78/10 to 2.94/10 (p<0.001)^24^; Body-based: Large effect on pain severity (ES=1.8)^27^; Pharmacological: Improved severity, interference, disability reduction (ES not reported)^27^
**Major Depressive Disorder (MDD)**	Total n = 493,281 N depression =376,872 N controls =116,409	Prevalence: 53.8^31^–65%^48^; Bidirectional Risk: OR=1.26^32^–1.88^33^; Common Types: Joint pain (37.8%)^31^, back pain (28.4%)^31^, widespread pain (1.9%)^34^	Female gender^34^, depression severity^35^, insomnia^32^, prolonged pain (>2 years)^35^, pain catastrophizing^35^, sedentary behavior (TV watching)^33^, White race (stronger in Black individuals)^34^ Protective: Employment^35^	Psychosocial (e.g., CBT)^25^, acupuncture + drugs^26^, CBT-based^27^, body-based^29^, pharmacological^27^	CBT: small effect (SMD=0.27, 95% CI –0.08–0.61)^25^; Acupuncture + drugs: Improved depression (MD=–2.95) and pain (MD=–1.06)^26^; CBT/body-based/pharmacological: Small to large effects on severity for MBIs (ES=0.32–0.86), interference (MD=–1.0), catastrophizing^27^
**Comorbid Depression and Anxiety**	Total n =15,212 N depression & anxiety =7,517 N controls =7,663	Prevalence: 50–60%^23^; Common Types: Low back pain, fibromyalgia^23^	None specifically identified	Mindfulness-Based Intervention (MBI)^27^	MBI: No significant pain severity reduction, large decrease in pain interference^27^
**Bipolar Disorder**	Total n =12,405894 N BD= 20,602 N controls =12,204,292	Prevalence: 23.7^22-^61.5%^48^; Relative Risk: RR=2.14 (95% CI 1.67–2.75)^22^; Common Types: Clinical pain across multiple sites^22^	Older age^36^, female gender^36^, White race^36^	None specifically reported	No specific outcomes reported
**Schizophrenia**	Total n=4,501,993 N schizophrenia =242,782 N controls =4,259211	Prevalence: 29.5^29^–47.2%^48^; Relative Risk: RR=1.65 (95% CI 0.68–3.99)^29^; Common Types: Head/face/mouth (33.3%)^37^	None specifically identified	None specifically reported	No specific outcomes reported
**ADHD**	Total n =8,463 N ADHD =263 N controls =8,200	Prevalence: 29.1–66.9%^28,42^; Common Types: Musculoskeletal, multisite pain^28^	Female gender^42^	None specifically reported	No specific outcomes reported
**ADHD and/or Autism**	Total n =77 N ADHD & autism =77	Prevalence: 76.6%^43^; Common Types: Chronic widespread pain (32.5%)^43^, lower back (46.8%)^43^, abdominal (29.9%)^43^, headaches (26%)^43^	None specifically identified; Protective: Stimulant use^43^	Stimulant use (noted as a factor, not a formal treatment study)^43^	Stimulants linked to reduced chronic widespread pain (16.7% vs. 42% without)^43^
**Dementia**	Total n=40 N dementia = 40	Prevalence: Not quantified^44^; Common Types: Not specified^44^	None specifically identified	Transcranial Direct Current Stimulation (tDCS)^44^	tDCS: Significant pain intensity reduction (d=0.69 NRS, d=1.12 MOBIDS)^44^
**Severe Mental Illness (SMI)**	Total n =116,783 N MDD =65,750 N BD= 38,117 N schizophrenia =12,916	Prevalence: 47.2–62.4%^48^ (MDD: 62.4%, BD: 61.5%, Schizophrenia: 47.2%); Common Types: Limb/extremity arthritis (22.8–32.3%)^48^, back (14.4–20.4%)^48^, abdominal/bowel (11.6–15.2%)^48^	None specifically identified	Opioid dispensing^48^	Opioids dispensed: 36.9% MDD, 45.5% BD, 27.2% schizophrenia; efficacy not detailed^48^

**Table 2.** Methodological quality of included systematic reviews using “A Measurement Tool to Assess Systematic Reviews” (AMSTAR) and AMSTAR +

References	Item 1	Item 2	Item 3	Item 4	Item 5	Item 6	Item 7	Item 8	Item 9	Item 10	Item 11	TOTAL AMSTAR	Item 1	Item 2	Item 3	Item 4	Item 5	Item 6	TOTAL AMSTAR +
Brandl et al. [23]	Y	N	Y	N	N	Y	Y	Y	Y	Y	Y	8/11	N/A	N	Y	N	Y	N	2/7
Rometsch-Ogioun et al. [24]	Y	N	Y	N	N	Y	Y	Y	Y	Y	Y	8/11	N/A	N/A	N/A	N/A	Y	N	1/7
Patel and Chrisinger [25]	Y	N/A	Y	N	Y	Y	Y	Y	Y	Y	Y	9/11	Y	N	N	N	N	N	2/7
You et al. [26]	Y	Y	Y	N	Y	Y	Y	Y	Y	Y	Y	10/11	Y	N	N	N	Y	N	3/7
Ma et al. [27]	Y	N	Y	Y	Y	Y	Y	Y	Y	Y	Y	10/11	N/A	N/A	N/A	N/A	N/A	N/A	0/7
Battison et al. [28]	Y	N	Y	N	Y	Y	N	Y	Y	N	Y	7/11	N/A	N/A	N/A	N/A	N/A	N/A	0/7
Stubbs et al. [29]	Y	N	Y	Y	Y	Y	Y	Y	Y	Y	Y	10/11	N	Y	N	N	Y	N	2/7
Stubbs et al. [22]	Y	N	Y	Y	Y	Y	Y	Y	Y	Y	Y	10/11	N	Y	N	N	Y	N	2/7

Y = yes; N = No; N/A = not applicable.

Detailed items of the AMSTAR:

1 - Was an ‘a priori’ design provided?

2 - Was there duplicate study selection and data extraction?

3 - Was a comprehensive literature search performed?

4 - Was the status of publication (that is gray literature) used as an inclusion criterion?

5 - Was a list of studies (included and excluded) provided?

6 - Were the characteristics of the included studies provided?

7 - Was the scientific quality of the included studies assessed and documented?

8 - Was the scientific quality of the included studies used appropriately in formulating conclusions?

9 - Were the methods used to combine the findings of studies appropriate?

10 - Was the likelihood of publication bias assessed?

11 - Was the conflict of interest stated?

Detailed items of the AMSTAR +:

1 - Was the majority of all meta-analysed studies double-blind? Was not assessed as not possible to realize in physical activity interventions (2 points)

2 - Was the total number of participants in the meta-analysis sufficiently large?

3 - Was the meta-analytically derived primary outcome result confirmed in at least one large study with approximately 100 patients per arm?

4 - Were studies with observed cases analyses included in the meta-analysis?

5 - Was the primary outcome result heterogeneous?

6 - Was there significant publication bias regarding the primary outcome result?

**Table 3.** Physiotherapy Evidence Database (PEDro) score for methodological quality assessment of randomized controlled trials investigating chronic pain in people with mental illness

Reference	Item 1	Item 2*	Item 3	Item 4	Item 5	Item 6	Item 7	Item 8	Item 9	Item 10	Item 11	Total score	Interpretation
Martorella et al. [44]	1	1	1	1	1	1	1	1	1	1	1	10/10	Excellent

Item 1 = eligibility criteria (does not contribute to the total score); item 2 = random allocation; item 3 = concealed allocation; item 4 = similar baseline; item 5 = subjected blinded; item 6 = therapists blinded; item 7 = assessors blinded; item 8 = < 15% dropouts; item 9 = intention-to-treat analysis; item 10 = between-group comparison; item 11 = point measures and variability data; 1 = described explicitly and in details; 0 = unclear, inadequately described. A total score of 9 to 10 was categorized as excellent quality, 6 to 8 as good quality, 4 to 5 as fair quality, and < 4 as poor quality.

**Table 4.** Newcastle – Ottawa Scale (NOS) for quality assessment of cohort studies

	Selection				Comparability	Outcome			
References	1. Representativeness of the exposed cohort	2. Selection of the non exposed cohort	3. Ascertainment of exposure	4. Demonstration that outcome of interest was not present at start of study	5. Comparability of cohorts on the basis of the design or analysis	6. Assessment of outcome	7. Was follow-up long enough for outcomes to occur	8. Adequacy of follow up of cohorts	Total
Rambla et al. [35]	1	0	1	1	0	1	1	1	7
Nicholl et al. [34]	1	0	1	0	0	1	0	0	3
Mundal et al. [42]	1	0	1	0	0	1	0	0	3
Asztély et al. [43]	1	0	1	0	0	1	1	0	5
Owen-Smith et al. [45]	1	1	1	1	1	1	0	0	6
Sepulveda-Torres et al. [37]	1	0	0	0	0	1	0	0	2
Shipherd et al. [30]	1	1	1	0	0	0	1	1	5
Hanseen et al. [31]	1	1	0	0	0	0	1	1	4
Trivedi et al.[36]	0	1	1	1	1	0	1	1	6
Zhu et al. [32]	1	0	1	0	0	1	1	0	4
Li et al. [33]	1	0	1	0	0	1	1	0	4

**Selection (Max: 4 stars)**

1. **Representativeness of the Exposed Cohort**: (a) Truly representative of the average (describe) in the community (*), (b) Somewhat representative of the average (describe) in the community, (c) Selected group (e.g., nurses, volunteers), (*d*) No description of the derivation of the cohort.

2. **Selection of the Non-Exposed Cohort**: (a) Drawn from the same community as the exposed cohort (*), (b) Drawn from a different source, (c) No description of the derivation of the non-exposed cohort.

3. **Ascertainment of Exposure**: (a) Secure record (e.g., surgical records) (*), (b) Structured interview (*), (c) Written self-report, (*d*) No description.

4. **Demonstration That Outcome of Interest Was Not Present at Start of Study**: (a) Yes, (b) No.

**Comparability (Max: 2 stars)**

1. **Comparability of Cohorts Based on Design or Analysis**: (a) Study controls for (select the most important factor)*(b) Study controls for any additional factor (). (This criterion could be modified to indicate specific control for a second important factor.)*

**Outcome (Max: 3 stars)**

1. **Assessment of Outcome**: (a) Independent blind assessment (*), (b) Record linkage (*), (c) Self-report, (*d*) No description.

2. **Was Follow-Up Long Enough for Outcomes to Occur?**: (a) Yes (select an adequate follow-up period for the outcome of interest) (*), (b) No.

3. **Adequacy of Follow-Up of Cohorts**: (a) Complete follow-up – all subjects accounted for (*), (b) Subjects lost to follow-up unlikely to introduce bias – small number lost – > (select an adequate %) follow-up, or description provided of those lost (*), (c) Follow-up rate < (select an adequate %) and no description of those lost, (*d*) No statement.

*Note*: A study can be awarded a maximum of one star for each numbered item within the Selection and Outcome categories. A maximum of two stars can be given for Comparability.

## Discussion

This umbrella review represents the first comprehensive synthesis of systematic reviews and meta-analyses examining chronic pain across mental disorders, consolidating evidence from 20 studies to address a critical yet underexplored intersection of psychiatry and physical health. Its significance lies in highlighting a pervasive comorbidity that exacerbates disability, escalates healthcare costs, and challenges conventional siloed treatment models, underscoring the need for integrated care approaches. The findings reveal chronic pain prevalence rates ranging from 23.7% in BD^30^ to 96% in PTSD [24], with elevated relative risks (e.g., RR = 1.65–2.14) across most conditions compared to the general population. However, treatment efficacy remains limited, with psychosocial interventions showing only modest effects and opioids remaining the dominant approach in SMI management. These results provide a foundation for a critical appraisal of the existing evidence and its clinical implications.

The intricate interplay between chronic pain and mental disorders presents a pressing clinical challenge, as reflected in the high prevalence rates and bidirectional risks identified in this review. Across anxiety disorders, depression, BD, schizophrenia, ADHD, autism, SMI, and dementia, chronic pain prevalence exceeded general population estimates of 20–25% [2, 3]. This synthesis confirms that individuals with mental disorders are disproportionately burdened by chronic pain, a finding with significant implications for psychiatric practice. However, methodological inconsistencies and therapeutic uncertainties temper these conclusions and highlight the need for a more nuanced understanding of this comorbidity.

For anxiety and stress-related disorders, particularly PTSD, the near-universal prevalence of chronic pain (88–96%) aligns with the role of trauma in amplifying somatic distress [46]. The elevated risk associated with the female gender and symptom severity [24] suggests the need for gender-sensitive screening and trauma-focused interventions. However, reliance on heterogeneous pain assessment methods limits confidence in these estimates.

In depression, with prevalence rates ranging from 53.8 to 65%, the bidirectional relationship with chronic pain is well-documented [32, 33], reinforcing neurobiological models of shared pathways, such as hypothalamic–pituitary–adrenal axis dysregulation and inflammatory cascades [47, 48]. While these findings underscore the necessity of integrated care models, the modest effects of psychosocial interventions [25] and the inconsistent efficacy of acupuncture [26] highlight a persistent therapeutic gap.

BD and schizophrenia present contrasting profiles in terms of chronic pain burden. In BD, prevalence rates range from 23.7% [22] to 61.5% [45], with increased opioid use [45] signaling a reliance on pharmacological management that risks exacerbating mood instability. This concern aligns with broader critiques of polypharmacy in mood disorders [49]. In schizophrenia, a pooled prevalence of 29.5% [29] is lower than in other SMIs, while an inconclusive relative risk estimate (RR = 1.65, 95% CI 0.68–3.99) challenges assumptions of a uniform pain burden across psychotic disorders. The wide CI and significant heterogeneity (*Q* = 389.48, *p* < 0.0001) suggest that study design differences—ranging from large administrative datasets [39] to smaller self-report studies [41]—play a role in these discrepancies. Additionally, under-detection due to altered pain perception, a phenomenon well-documented in schizophrenia [50], may contribute to these lower estimates.

The ADHD and autism-ADHD cohorts displayed unexpectedly high chronic pain prevalence (29.1–76.6%), with female sex consistently identified as a risk factor [42]. Stimulant use was linked to reduced pain in individuals with autism-ADHD overlap [43], an intriguing finding that warrants further exploration. However, given the limited number of studies available, the generalizability of these findings remains uncertain, and questions regarding the role of sensory processing differences in pain perception persist [49].

In SMI, encompassing MDD, BD, and schizophrenia, prevalence rates ranged from 47.2 to 62.4% [45], with notably high rates in MDD (62.4%) and BD (61.5%) compared to schizophrenia (47.2%). The reliance on opioids in SMI [45], with dispensing rates of 27.2–45.5% among those with chronic pain, underscores a critical gap in evidence-based, non-opioid pain management strategies [51]. The approach to chronic pain in dementia, which includes non-invasive neuromodulation techniques such as transcranial direct current stimulation [21], represents a rare positive development, although the absence of robust prevalence data limits broader conclusions.

It is worth noting that, despite inconsistent improvements in pain severity across intervention studies included in this review, several studies (e.g., those examining CBT) reported significant reductions in pain-related interference following treatment. Given that pain interference contributes to the relationship between pain severity, functional impairment, and perceived disability [52], reductions in interference, such as improved ability to engage in daily activities, may represent meaningful clinical gains even in the absence of changes in pain intensity. Interventions targeting psychosocial factors that enhance pain-related functioning may therefore contribute to improved quality of life and perceived disability, further supporting their therapeutic value.

Clinicians should adopt a proactive, interdisciplinary approach to managing chronic pain in individuals with mental disorders, integrating routine pain assessment into psychiatric evaluations using validated tools such as the Brief Pain Inventory [53] or the McGill Pain Questionnaire [54]. Given the high prevalence and bidirectional nature of this comorbidity, mental health practitioners must be trained to recognize pain as a distinct and treatable condition rather than attributing it solely to psychiatric symptoms. Given concerns over opioid reliance, particularly evident in SMI populations where up to 45.5% of BD patients with chronic pain receive opioids [45], clinicians should consider safer pharmacological alternatives with both analgesic and psychotropic benefits, such as serotonin-norepinephrine reuptake inhibitors or atypical antipsychotics with emerging evidence for pain modulation. Collaborative care models integrating pain specialists, physiotherapists, and mental health professionals should be implemented to enhance patient-centered care, particularly for individuals with SMI who face heightened treatment barriers. To improve long-term outcomes, clinicians should advocate for structural changes in healthcare settings that facilitate integrated care, such as co-located psychiatric and pain management services, while leveraging digital health tools to monitor pain symptoms remotely. Finally, the current review revealed pain disparities by sex and ethnic background, with *female sex and White ethnicity* emerging as key risk factors for experiencing more severe pain. This is consistent with prior research showing that women tend to report more intense pain experiences [55], and that ethnic minority groups may be at a relative advantage compared to White individuals, particularly when socioeconomic status (SES) is taken into account [56]. However, in the context of mental illness, *diagnostic overshadowing*, where physical symptoms are misattributed to psychiatric conditions [57], may further compound these disparities. In particular, the intersection of *female sex* [58] *and low SES* [59] may exacerbate barriers to appropriate pain assessment and treatment, contributing to broader healthcare access inequalities. Therefore, addressing disparities in access to pain management, particularly for marginalized populations with mental disorders, should be a public health priority, ensuring equitable delivery of evidence-based interventions.

Therapeutic implications are equally complex. Although interventions such as acupuncture [27] and transcranial direct current stimulation [44] show promise, their applicability remains narrow. Psychosocial interventions, particularly CBT, yield at best small-to-moderate effects [25, 27], and opioid use in SMI populations [45] raises concerns in the context of the global opioid crisis [60]. The phenomenon of diagnostic overshadowing, where pain symptoms are misattributed to psychiatric illness [61], further complicates management. This issue is particularly problematic in schizophrenia, where delayed pain detection may contribute to worse health outcomes [50].

### Future research

Future research must prioritize the standardization of chronic pain assessment in psychiatric populations through validated tools that integrate subjective and objective measures, ensuring consistency across studies. Longitudinal cohort studies are needed to establish causal pathways between chronic pain and mental disorders, incorporating biological markers such as inflammatory cytokines and neuroendocrine dysfunction to clarify mechanistic underpinnings. Expanding research on underexplored conditions, including ADHD, autism, and dementia, while identifying additional risk factors such as socioeconomic status and lifestyle behaviours, will enhance targeted prevention strategies. Given the limited efficacy of current interventions and the overreliance on opioids, particularly in SMI where opioid dispensing is prevalent [49], randomized controlled trials should evaluate multimodal treatment approaches, including pharmacological and non-pharmacological synergies, neuromodulation techniques such as transcranial direct current stimulation and transcranial magnetic stimulation, as well as sleep and structured exercise interventions that target both pain modulation and mental health outcomes. Investigating the optimal type, intensity, and duration of exercise targeting chronic pain for different psychiatric populations, alongside its neurobiological and psychological mechanisms, will be crucial for integrating exercise into standard care. Addressing structural barriers to integrated pain-psychiatry care requires pragmatic implementation research to assess the feasibility of interdisciplinary models, with a focus on accessibility for marginalized populations. Additionally, due to substantial methodological heterogeneity across included reviews (e.g., various focuses on different types of pain, outcome measures, intervention types), quantitative synthesis (e.g., meta-meta-analytical pooling or network meta-analysis) was deemed inappropriate for this umbrella review. However, certain areas, such as pharmacological treatments (e.g., stimulants and antidepressants) for patients with ADHD, autism, and SMI in general, warrant future quantitative synthesis once a sufficient number of high-quality and homogenously reported studies become available. Future systematic reviews and meta-analyses must also adopt rigorous methodologies, including standardized inclusion criteria, individual participant data analyses, and network meta-analyses, to refine prevalence estimates and optimize treatment comparisons. By advancing these research priorities, the field can move beyond documenting high chronic pain prevalence in psychiatric populations toward developing evidence-based, personalized, and accessible interventions that improve clinical outcomes and quality of life.

### Strengths and limitations

This review’s strengths lie in its status as the first umbrella review to synthesize top-tier evidence across a broad spectrum of mental disorders, providing a rigorous assessment of chronic pain prevalence, risk factors, and treatment approaches. Its inclusion of diverse populations, from PTSD to dementia, and large sample sizes (up to 12,375,644 participants) across 957,168 cases and 16,606,910 controls enhances its clinical relevance. However, significant limitations remain, including inconsistent pain assessment methods, unclear symptom duration criteria, and study heterogeneity. Publication bias may have influenced the evidence base, given systematic reviews and meta-analyses are more likely to include studies reporting statistically significant findings. Although we aimed to minimize overlap across reviews by prioritizing the most recent and comprehensive reviews and excluding primary research already incorporated within these reviews, there remains a risk of double-counting, particularly in areas with a high volume of publications (e.g., depression, PTSD, and chronic pain). Finally, our review did not retrieve any studies focusing specifically on chronic pain in people with personality disorders. This is not only a limitation of the current review but also reflects a critical gap in current evidence base, despite the clinical relevance of personality disorders in this context. Individuals with personality disorders, especially borderline personality disorder, have been reported to experience increased pain severity and unique pain presentation (e.g., central sensitization) [62, 63]. These shortcomings highlight the urgent need for methodological refinement in future research.

In conclusion, this review underscores the widespread burden of chronic pain in mental disorders while exposing a critical lack of integrated treatment approaches. Addressing this challenge requires the implementation of standardized pain assessments in psychiatric practice and the development of evidence-based, multidisciplinary treatment strategies that bridge the divide between pain management and mental health care.

## Supporting information

10.1192/j.eurpsy.2025.10074.sm001 Stubbs et al. supplementary material 1 Stubbs et al. supplementary material

10.1192/j.eurpsy.2025.10074.sm002 Stubbs et al. supplementary material 2 Stubbs et al. supplementary material

## Data Availability

The data that support the findings of this study are available from the corresponding author upon reasonable request.

## Supplementary material

The supplementary material for this article can be found at http://doi.org/10.1192/j.eurpsy.2025.10074.
