# Supplementary material for: Chronic pain in mental disorders: An umbrella review of the prevalence, risk factors, and treatments across 957,168 people with mental disorders and 16,606,910 controls
Source: Eur Psychiatry. 2025 Aug 12;68(1):e113. doi: 10.1192/j.eurpsy.2025.10074 (PMC12438992; doi:10.1192/j.eurpsy.2025.10074)
Supplement: Stubbs et al. supplementary material 2 — Stubbs et al. supplementary material [file S0924933825100746sup002.docx]

**Table 1:** Synthesis of chronic pain prevalence, risk factors and treatments by mental disorders.

| **Mental Disorder** |  |  |  | **Sample size** | **Pain characteristics** | **Primary risk factors** | **Treatment approaches** | **Treatment outcomes** |
| --- | --- | --- | --- | --- | --- | --- | --- | --- |
| **Anxiety and Stress Disorders (PTSD)** |  |  |  | Total n =22,335  N PTSD =11,200  N controls =11,135 | Prevalence: 66^30^–96%^24^;  Common Types: Stomach pain (87.7%)^24^, chest pain (84.4%)^24^, back pain (82.2%)^24^, arms/legs (85.0%)^24^, headaches (85.7%)^24^ | Older age^24^, female gender^24^, socioeconomic difficulties^24^, severe PTSD symptoms^24^ | Emotional Freedom Technique (EFT)^24^, body-based interventions^27^, pharmacological therapy^27^ | EFT: Reduced pain scores from 4.78/10 to 2.94/10 (p<0.001)^24^; Body-based: Large effect on pain severity (ES=1.8)^27^; Pharmacological: Improved severity, interference, disability reduction (ES not reported)^27^ |
| **Major Depressive Disorder (MDD)** |  |  |  | Total n = 493,281  N depression =376,872  N controls =116,409 | Prevalence: 53.8^31^–65%^48^; Bidirectional Risk: OR=1.26^32^–1.88^33^; Common Types: Joint pain (37.8%)^31^, back pain (28.4%)^31^, widespread pain (1.9%)^34^ | Female gender^34^, depression severity^35^, insomnia^32^, prolonged pain (>2 years)^35^, pain catastrophizing^35^, sedentary behavior (TV watching)^33^, White race (stronger in Black individuals)^34^  Protective: Employment^35^ | Psychosocial (e.g., CBT)^25^, acupuncture + drugs^26^, CBT-based^27^, body-based^29^, pharmacological^27^ | CBT: small effect (SMD=0.27, 95% CI -0.08–0.61)^25^; Acupuncture + drugs: Improved depression (MD=-2.95) and pain (MD=-1.06)^26^; CBT/body-based/pharmacological: Small to large effects on severity for MBIs (ES=0.32-0.86), interference (MD=-1.0), catastrophizing^27^ |
| **Comorbid Depression and Anxiety** |  |  |  | Total n =15,212  N depression & anxiety =7,517  N controls =7,663 | Prevalence: 50–60%^23^; Common Types: Low back pain, fibromyalgia^23^ | None specifically identified | Mindfulness-Based Intervention (MBI)^27^ | MBI: No significant pain severity reduction, large decrease in pain interference^27^ |
| **Bipolar Disorder** |  |  |  | Total n =12,405894  N BD= 20,602  N controls =12,204,292 | Prevalence: 23.7^22-^61.5%^48^; Relative Risk: RR=2.14 (95% CI 1.67–2.75)^22^;  Common Types: Clinical pain across multiple sites^22^ | Older age^36^, female gender^36^, White race^36^ | None specifically reported | No specific outcomes reported |
| **Schizophrenia** |  |  |  | Total n=4,501,993  N schizophrenia =242,782  N controls =4,259211 | Prevalence: 29.5^29^–47.2%^48^; Relative Risk: RR=1.65 (95% CI 0.68–3.99)^29^;  Common Types: Head/face/mouth (33.3%)^37^ | None specifically identified | None specifically reported | No specific outcomes reported |
| **ADHD** |  |  |  | Total n =8,463  N ADHD =263  N controls =8,200 | Prevalence: 29.1–66.9%^28,42^; Common Types: Musculoskeletal, multisite pain^28^ | Female gender^42^ | None specifically reported | No specific outcomes reported |
| **ADHD and/or Autism** |  |  |  | Total n =77  N ADHD & autism =77 | Prevalence: 76.6%^43^; Common Types: Chronic widespread pain (32.5%)^43^, lower back (46.8%)^43^, abdominal (29.9%)^43^, headaches (26%)^43^ | None specifically identified; Protective: Stimulant use^43^ | Stimulant use (noted as a factor, not a formal treatment study)^43^ | Stimulants linked to reduced chronic widespread pain (16.7% vs. 42% without)^43^ |
| **Dementia** |  |  |  | Total n=40  N dementia = 40 | Prevalence: Not quantified^44^;  Common Types: Not specified^44^ | None specifically identified | Transcranial Direct Current Stimulation (tDCS)^44^ | tDCS: Significant pain intensity reduction (d=0.69 NRS, d=1.12 MOBIDS)^44^ |
| **Severe Mental Illness (SMI)** |  |  |  | Total n =116,783  N MDD =65,750  N BD= 38,117  N schizophrenia =12,916 | Prevalence: 47.2–62.4%^48^ (MDD: 62.4%, BD: 61.5%, Schizophrenia: 47.2%); Common Types: Limb/extremity arthritis (22.8–32.3%)^48^, back (14.4–20.4%)^48^, abdominal/bowel (11.6–15.2%)^48^ | None specifically identified | Opioid dispensing^48^ | Opioids dispensed: 36.9% MDD, 45.5% BD, 27.2% schizophrenia; efficacy not detailed^48^ |

**Supplementary Table 1.** Full search terms for literature search

**Date of Search:** 1st February 2025
**Inclusion Criteria:** Studies published between 1st January 2000 and 1st February 2025
**Databases Searched:** MEDLINE (via PubMed), PsycINFO, Embase, Web of Science, and CINAHL

| **Database** | **Exact Search String** |
| --- | --- |
| **MEDLINE (via PubMed)** | ("mental disorders"[MeSH Terms] OR "mental illness" OR "mental health disorder*" OR "psychiatric disorder*" OR "depression" OR "major depressive disorder" OR "bipolar disorder" OR "schizophrenia" OR "psychosis" OR "anxiety disorder*" OR "post-traumatic stress disorder" OR "ADHD" OR "autism" OR "autism spectrum disorder" OR "dementia" OR "cognitive disorder*") AND ("chronic pain"[MeSH Terms] OR "chronic pain" OR "persistent pain" OR "musculoskeletal pain" OR "somatic pain") AND ("systematic review"[Publication Type] OR "meta-analysis"[Publication Type] OR "systematic review" OR "meta-analysis") AND ("2000/01/01"[Date - Publication] : "2025/02/01"[Date - Publication]) |
| **Embase** | ('mental disease'/exp OR 'mental illness' OR 'depression'/exp OR 'schizophrenia'/exp OR 'bipolar disorder'/exp OR 'attention deficit hyperactivity disorder'/exp OR 'autism spectrum disorder'/exp OR 'dementia'/exp) AND ('chronic pain'/exp OR 'persistent pain' OR 'musculoskeletal pain'/exp OR 'somatic pain') AND ('systematic review'/de OR 'meta analysis'/de OR 'systematic review' OR 'meta-analysis') AND [2000-2025]/py |
| **PsycINFO** | (DE "Mental Disorders" OR "mental illness" OR "psychiatric disorder*" OR "depression" OR "bipolar disorder" OR "schizophrenia" OR "psychosis" OR "anxiety disorder*" OR "PTSD" OR "ADHD" OR "autism" OR "autism spectrum disorder" OR "dementia") AND ("chronic pain" OR "persistent pain" OR "musculoskeletal pain" OR "somatic pain") AND ("systematic review" OR "meta-analysis") AND (PY >= 2000 AND PY <= 2025) |
| **CINAHL (via EBSCOhost)** | (MH "Mental Disorders+" OR "mental illness" OR "depression" OR "bipolar disorder" OR "schizophrenia" OR "autism" OR "dementia") AND ("chronic pain" OR "persistent pain" OR "musculoskeletal pain") AND (MH "Systematic Review" OR "systematic review" OR "meta-analysis") AND (Published Date: 20000101 - 20250201) |
| **Web of Science (Core Collection)** | TS=("mental illness" OR "mental disorder*" OR "psychiatric disorder*" OR "depression" OR "bipolar disorder" OR "schizophrenia" OR "ADHD" OR "autism" OR "dementia") AND TS=("chronic pain" OR "persistent pain" OR "musculoskeletal pain") AND TS=("systematic review" OR "meta-analysis") AND PY=(2000-2025) |

**Supplementary Table 2.** Glossary of Key Pain Constructs

| **Term** | **Definition** | **Source / Note** |
| --- | --- | --- |
| **Localized Pain** | Consistent and maximum pain limited to a single region or body site. | Mick G, Baron R, Finnerup NB, et al. What is localized neuropathic pain? A first proposal to characterize and define a widely used term. Pain Manag. 2012;2(1):71–77 |
| **Multisite Pain** | Pain that occurs simultaneously at more than 1 region or body site | Carnes D, Parsons S, Ashby D, et al. Chronic musculoskeletal pain rarely presents in a single body site: results from a UK population study. Rheumatology (Oxford) 2007;46(7):1168–1170. |
| **Chronic widespread Pain** | Chronic pain present on both side of the body, above and below the waist and in the spine | Rahman (2022). Chronic widespread pain and the fibromyalgia syndrome. Medicine , 50(3): 184-188 |
| **Chronic regional pain** | Chronic pain characterised by hyperalgesia and allodynia, frequently involving limbs and developed after trauma or surgery | Ott S, Maihöfner C. Signs and symptoms in 1,043 patients with complex regional pain syndrome. J Pain. 2018;19(6):599–611 |
| **Low back pain** | Non-radiating pain affecting primarily in the back | Maher C., Underwood M., Buchbinder R. Non-specific low back pain. Lancet. 2017;389:736–747 |
| **Musculoskeletal pain** | Acute or recurrent pain in the bones, muscles, joints, tendons and nerves | Smith E, Hoy DG, Cross M, et al. The global burden of other musculoskeletal disorders: estimates from the Global Burden of Disease 2010 study. Ann Rheum Dis. 2014;73:1462–1469 |
| **Arthritis** | Acute or chronic joint inflammation | Ma L, Cranney A, Holroyd-Leduc JM. Acute monoarthritis: what is the cause of my patient's painful swollen joint? CMAJ. 2009;180(1):59-65. |
| **Osteoarthritis** | Characterised by articular cartilage degeneration and persistent pain | Jang S., Lee K., Ju J.H. Recent Updates of Diagnosis, Pathophysiology, and Treatment on Osteoarthritis of the Knee. Int. J. Mol. Sci. 2021;22:2619. |
| **Fibromyalgia** | Characterised by widespread pain and muscle tenderness, along with fatigue, cognitive dysfunction and somatic complaints | Wolfe F, Clauw DJ, Fitzcharles MA, et al. The American College of Rheumatology preliminary diagnostic criteria for fibromyalgia and measurement of symptom severity. Arthritis Care Res (Hoboken). 2010;62(5):600–610. |
| **Pain Severity** | A subjective rating of pain intensity, often measured using scales such as the Numeric Rating Scale (NRS) or Visual Analogue Scale (VAS). | Dijkers M. (2010). Comparing quantification of pain severity by verbal rating and numeric rating scales. The journal of spinal cord medicine, *33*(3), 232–242. |
| **Pain interference** | The degree to which pain interferes with an individual’s daily activities | Riley WT, Rothrock N, Bruce B, Christodolou C, Cook K, Hahn EA, et al. Patient-reported outcomes measurement information system (PROMIS) domain names and definitions revisions: Further evaluation of content validity in IRT-derived item banks. Qual Life Res. 2010;19(9):1311–21 |
| **Pain catastrophizing** | The tendency to magnify the threat value of pain, feel helplessness and an inability to inhibit pain—related thoughts | Quartana, P. J., Campbell, C. M., & Edwards, R. R. (2009). Pain catastrophizing: a critical review. Expert review of neurotherapeutics, *9*(5), 745–758 |

**Supplementary Table 3.** Prevalence and risk of chronic pain across mental disorders

| **First author + year** | **Setting** | **Study Design** | **Sample** | **Chronic Pain type/location** | **Chronic pain assessment method** | **Mental illness diagnostic criteria** | **Prevalence**  **/risk** |
| --- | --- | --- | --- | --- | --- | --- | --- |
| **Anxiety and stress disorders** | | | | | | | |
| Shipherd 2007 | Specialist medical center for PTSD | Observational | N=85 | Various: all forms of arthritis, persistent headaches, fibromyalgia, polyarthralgia, chronic back pain and joint pain (including ankle, knee, shoulder or wrist) | ICD | Medical records, involved a clinical interview and administration of a series of questionnaires to confirm | 56 (66%) had a chronic pain diagnosis   - 16 with chronic low back pain - 14 had general chronic pain, secondary to other conditions (diabetic neuropathy, lymphoma, emphysema, prostate cancer, coronary heart disease, hepatitis C and/or hypertension) - 8 with osteoarthritis - 4 persistent headache - 5 joint pain - 2 chest pain (chronic obstructive pulmonary disease and cardiac stent, gastro reflux) - 2 neck injury - 2 arthralgia/sciatica - 1 fibromyalgia - 1 carpel tunnel syndrome - 1 degenerative disk disorder |
| Rometsch-Ogioun El Sount 2019 | Multiple:  psychiatric trauma clinics +  medical  /psychiatric treatment centers +  rehabilitation centers +  community settings | SR & MA | K=15  n PTSD = 10,931 vs n controls = 11,135 | Various chronic pain sites:  headaches, back pain, pain in arms and legs, pelvic pain, stomach pain, joint pain, chest pain | Various, validated scales, clinical assessments | DSM / ICD | 88-96% had chronic pain  Common sites were:   - Stomach pain: 87.7% - Chest pain: 84.4% - Arms/legs pain: 85% - Back pain: 82.2% - Head pain: 85.7% |
| **Depression** | | | | | | | |
| Hanssen 2018 | Community and secondary health care  general practitioners out- and inpatient clinics | Observational | N=102 older patients with medically unexplained symptoms  N=144 older medially explained symptoms-patients  N=275 MDD patients | Multiple sites: back, neck, abdominal, joint and chest pain, headache/migraine, and face-ache lasted for 90 days or more in the past 6 months | Graded Chronic Pain Scale and usage of pain medications  Chronic pain was defined as present for 90 days or more in the past 6 months | DSM | Chronic pain: 53.8% (n=148) among older people with MDD   - Back pain: 28.4% 9n=42) - Neck pain: 11.5% (n=17) - Headache/migraine: 6.1% (n=9) - Face-ache: 1% (n=3) - Abdominal pain: 9.5% (n=14) - Joint pain: 37.8% (n=56) - Chest pain: 4.7% (n=7) |
| Zhu 2024 | Community | Mendelian randomisation | n=341,797 | Chronic regional pain (limb, back, neck, head and abdomen pain) and fibromyalgia | ICD | Structured diagnostic interviews, electronic medical records or self-reported diagnosis or treatment for clinical depression by a medical professional | MDD associated with a higher risk of chronic regional pain (OR=1.26, 95% CI 1.16-1.38) |
| Li 2024 | Community | Mendelian randomisation | N=225 SNPs for multi-site chronic pain  N=69 SNPs for MDD | Multiple | The sum of body sites where multiple-site chronic pain lasting at least 3 months | DSM or ICD | Multiple-site chronic pain was associated with a higher risk of MDD (OR=1.88, 95% CI 1.64-2.15) |
| **Comorbid depression and anxiety** | | | | | | | |
| Brandl 2022 | Multiple settings | SR & MA | K = 320  n MDD = 5,248 vs n controls = 5,425  N ANX = 2,269 vs n controls = 2,238 | Various chronic pain conditions (low back pain, fibromyalgia) and locations | Multiple, but all chronic pain | DSM | Comorbidity rates with MDD/ANX = 50-60% |
| **Affective disorders** | | | | | | | |
| Nicholl 2014 | Community | Observational | N=149,611 | 7 specific sites including headache, facial, neck or shoulder, back, stomach or abdominal, hip and knee and pain all over the body | Pain at each site had been present for more than 3 months were defined as having chronic pain  Chronic multisite pain was defined if reported chronic pain in two body sites or more | Brief form ICD | Depression:   - 7,736 (24.3%) 1 site chronic pain - 6,299 (19.8%) 2-3 sites chronic pain - 1,416 (4.5%) 4-7 sites chronic pain - 597 (1.9%) widespread pain   Bipolar:   - 373 (45.2%) 1 site chronic pain - 365 (22.6%) 2-3 sites chronic pain - 93 (5.8%) 4-7 sites chronic pain - 53 (3.3%) widespread pain   Compared to non-mood disordered group, among bipolar disorder group:   - 2-3 cites relative risk ratio (RRR) 1.84 (95% CI 1,61, 2.11) - 4-7 sites RRR 2.39 (95% CI 1.88, 3.03) - widespread pain RRR 2.37 (95% CI 1.73, 3.23)   Compared to non-mood disordered group, among MDD group   - 2-3 cites relative risk ratio (RRR) 1.59 (95% CI 1,54, 1.65) - 4-7 sites RRR 2.13 (95% CI 1.98, 2.30) - widespread pain RRR 1.86 (95% CI 1.66, 2.08) |
| **Schizophrenia** | | | | | | | |
| Sepulveda-Torres 2021 | Public hospital | Cross-sectional | 79 | Mixed | McGill Pain Questionnaire (MPQ) | CIE-10 | - Abdominal: 8.3% (n=1) - Head, face, mouth: 33.3% (n=4) - Lumbar, sacral, and coccyx: 16.7% (n=2) - Lower limbs: 16.7% (n=2) - Shoulders and upper limbs: 8.3% (n=1) - Cervical: 16.7% (n=2) |
| Stubbs et al 2014 | Multiple | Meta-analysis | N=94,249 (schizophrenia) vs. n=4,247,724 (controls) | Mixed (various body sites) | Medical records, self-reports (>3 months), questionnaires | ICD-9, ICD-10, DSM-IV | Pooled prevalence: 29.5% (95% CI 7.4–51.6%) RR vs. controls: 1.65 (95% CI 0.68–3.99) |
| **Bipolar disorder** | | | | | | | |
| Stubbs 2015 | Multiple | Meta-analysis | N=12,375,644 individuals (BD n=171,352, n controls=12,204,292) | Clinical pain across multiple body sites general | Mixed | Diagnostic criteria (e.g. DSM-IV or ICD-10), valid screening measures (e.g. Alcohol Use Disorder and Associated Disabilities Interview Schedule—DSM-IV Version) or medical record | Pooled prevalence of chronic pain in 106,214 = 23.7% [95% CI = 13.1–36.3%  RR versus controls 2.14 [95% CI = 1.67–2.75%, (df = 7) |
| **ADHD** | | | | | | | |
| Battison 2023 | Various settings across 6 countries | Scoping review | K = 11 | Musculoskeletal pain, abdominal pain, complex regional Pain syndrome,  joint pain associated with hypermobility, multi-site pain | Clinical diagnoses of chronic pain >3 months or ICD | DSM or ICD | 29.1-66.9% |
| Mundal 2024 | Community health survey | 9-year longitudinal study with 3 time points | n ADHD = 263  vs 8,200 controls | Multiple sites including head, neck, upper/lower back, chest, gastrointestinal, arms, legs | Self-report chronic pain >3 months once at least once a week | DSM/ICD | 66.5% in ADHD vs 44.5% controls |
| **ADHD and/or autism** | | | | | | | |
| Asztély 2019 | Child Neuropsychiatric Clinic | observational | n =77  n=46 ASD with or without ADHD and n=46 ADHD only | Chronic widespread pain and chronic regional pain  Multiple sites: chest, neck, shoulder, arm-hand, thoracic spine, lumbar spine, hip-thigh, knee, calf-foot, stomach and head | Experienced pain for more than 3 months during the last 12 months | DSM | - Chronic pain: 76.6% - Chronic widespread pain: 32.5%   - Autism: 23.5%   - ADHD: 39%   Among the whole group:   - Lower back: 46.8% - Recurrent headache: 26% - Abdominal: 29.9% pain and - Abdominal + headache: 14.3% |
| **Severe mental illness** | | | | | | | |
| Owen-Smith 2020 | Medical records from 13 Mental Health Research Network | Longitudinal | Patients with MDD  (N = 65,750), Patients with Bipolar Disorder  (N = 38,117), Patients with Schizophrenia  (N = 12,916) | Mixed | NDC codes | ICD-10 | **MDD**   - Any: 62.4% (n=41,036) - Back: 20.4% (n=13,419) - Neck: 10.5% (n=6,877) - Limb /extremity, arthritis: 32.3% (n=21,239) - Fibromyalgia or widespread muscle pain: 6.5% (n=4,262) - Headache: 12.7% (n=8,359) - Orofacial /ear/ temporomandibular: 1.1% (n=728) - Abdominal/bowel: 15.1% (n=9,922) - Chest: 7.6% (n=4,995) - Urogenital / pelvic / menstrual: 4.9% (n=3,222) - Fractures / contusions / sprains / strains: 13.0% (n=8,542) - Other painful conditions: 12.2% (n=7,994)   **BPD:**   - Any: 61.5% (n=23,423) - Back: 20.4% (n=7,756) - Neck: 9.7% (n=3,713) - Limb / extremity, arthritis: 31.6% (n=12,052) - Fibromyalgia / widespread muscle pain: 6.3% (n=2,384) - Headache: 13.1% (n=5,000) - Orofacial / ear / temporomandibular pain: 1.3% (n=477) - Abdominal / bowel: 15.2% (n=5,777) - Chest: 7.9% (n=3,009) - Urogenital / pelvic/menstrual: 5.1% (n=1,925) - Fractures / contusions / sprains / strains: 14.6% (n=5,567) - Other painful conditions: 10.9% (n=4,137 )   **Schizophrenia:**   - Any: 47.2% (n=6,092) - Back: 14.4% (n=1,855) - Neck: 5.8% (n=754) - Limb/extremity, arthritis: 22.8% (n=2,942) - Fibromyalgia / widespread muscle pain: 3.0% (n=386) - Headache: 7.5% (n=973) - Orofacial / ear / temporomandibular: 0.9% (n=112) - Abdominal / bowel: 11.6% (n=1,497) - Chest: 7.6% (n=975) - Urogenital / pelvic / menstrual: 2.2% (n=280) - Fractures / contusions / sprains / strains: 10.8% (n=1,392) - Other painful conditions: 8.7% (n=1,117 ) |

**Supplementary Table 4.** Risk factors for chronic pain across mental disorders

| **First author + year** | **Setting** | **Study Design** | **Sample Size**  **[N studies**  **n participants]** | **Chronic Pain type/location** | **Chronic pain assessment method** | **Mental illness diagnostic criteria** | **Risk factors** |
| --- | --- | --- | --- | --- | --- | --- | --- |
| **Anxiety and stress disorders** | | | | | | | |
| Rometsch-Ogioun El Sount 2019 | Multiple:  psychiatric trauma clinics +  medical  /psychiatric treatment centers +  rehabilitation centers +  community settings | SR & MA | K =15  n PTSD = 10,931 vs n controls = 11,135 | Various chronic pain sites:  headaches, back pain, pain in arms and legs, pelvic pain, stomach pain, joint pain, chest pain | Various, validated scales, clinical assessments | DSM / ICD | Increasing age (β = 0.206, p = .008); female gender (β = 0.206, p = .008); living difficulties (β = 0.159, p = .047); more severe PTSD symptoms (β = 0.217-0.277, p = .028-.005) |
| **Depression** | | | | | | | |
| Nicholl 2014 | Community | Observational | N=144,139 | 7 specific sites including headache, facial, neck or shoulder, back, stomach or abdominal, hip and knee and pain all over the body | Pain at each site had been present for more than 3 months were defined as having chronic pain  Chronic multisite pain was defined if reported chronic pain in two body sites or more | Brief form ICD | Comorbid chronic pain and depression was more common in the White participants (n=14,998, 11.1%) than black (n=330, 8.5%) and Asian (n=340, 7.5%) participants  Compared to participants without chronic pain, the association between depression and chronic pain was strongest for black participants: OR 1.86 (95% CI 1.52-2.27) for presence of chronic pain, 1 site RRR=1.49 (95% CI 1.16, 1.91). 2-3 sites RRR 1.98 (95% CI 1.53,2.56), 4-7 sites RRR=3.23 (95% CI 2.09-4.99), pain all over the body RRR=3.31 (95% CI 2.05, 5.33) |
| Zhu 2024 | Community | Mendelian randomisation | n=341,797 | Chronic regional pain (limb, back, neck, head and abdomen pain) and fibromyalgia | ICD-10 | Structured diagnostic interviews, electronic medical records or self-reported diagnosis or treatment for clinical depression by a medical professional | Insomnia (effect =1.04) for chronic regional pain |
| Rambla 2023 | Primary care centers in the province of Tarragona, Catalonia, Spain | Secondary analysis of a RCT | n = 317 | Spine, limb or both | Moderate or severe chronic musculoskeletal pain via Brief Pain Inventory Pain severity scale ≥ + more than 3 months | DSM | Baseline pain severity predicted pain severity at 3 (β=0.53, 95% CI 0.37-0.68) and 12 months (β=0.48, 95% CI 0.29-0.67), and interference at 3 (β=0.26, 95% CI 0.10-0.42) and 12 months (β=0.20, 95% CI 0,02-0.39)  Pain >2 years of evolution predicted long-term pain severity (β=0.91, 95% CI 0.11-1.71), greater severity (β=0.91, 95% CI 0.11-1.71) and interference (β=1.23, 95% 0.41-2.04) at 12 months  Baseline pain interference predicted interference at 3 (β=0.27, 95% CI 0.11-0.43) and 12 month (β=0.21, 95% CI 0.03-0.40)  Depression severity predicted more interference at 12 months (β=0.58, 95% CI 0.04-1.11)  Occupation status as active worker predicted less interference at 3 (β=-0.74, 95% CI -1.36, -0.13) and 12 months (β=-0.96, 95% CI -1.71, -0.21)  Currently working predicts less pain severity at 12 months (β=-0.77, 95% CI 1.52, 0.02)  Pain catastrophising predicted pain severity (β=0.03, 95% CI 0.00-0.05) and interference (β=0.03, 95% CI 0.00-0.05) at 3 months only |
| Li 2024 | Community | Mendelian randomisation | 225 SNPs for multi-site chronic pain, 69 SNPs for MDD | Multiple | The sum of body sites where multiple-site chronic pain lasting at least 3 months | DSM or ICD | Sedentary behavior: time spent on watching tv (OR=1.46, 95% CI 1.39-1.53); using computer (OR=0.88, 95% CI 0.83-0.93) associated with chronic pain. |
| **Bipolar disorder** | | | | | | | |
| Trivedi 2022 | Hospital records | Cross-sectional | 15,125 BP patients with CP and 15,125 BP patients without CP | Unspecified | ICD-10 | ICD-10 | Compared with BD without CPD, most patients in the BD with CPD group were older (mean age, 47.6 vs. 40.4 years), female (58.4% vs. 55.2%), and white (77.2% vs. 66.7%). |
| **ADHD** | | | | | | | |
| Mundal 2024 | Community health survey | 9-year longitudinal study with 3 time points | n ADHD = 263  vs 8,200 controls | Multiple sites including head, neck, upper/lower back, chest, gastrointestinal, arms, legs | Self-report chronic pain >3 months once at least once a week | DSM/ICD | female sex |
| **ADHD and/or autism** | | | | | | | |
| Asztély 2019 | Child Neuropsychiatric Clinic | Observational | n =77  46 ASD with or without ADHD and 46 ADHD only | Chronic widespread pain and chronic regional pain  multiple sites: chest, neck, shoulder, arm-hand, thoracic spine, lumbar spine, hip-thigh, knee, calf-foot, stomach and head | Experienced pain for more than 3 months during the last 12 months | DSM | No significant differences between those with ASD and ADHD as main diagnosis in terms of abdominal pain (29.4% and 31.7% respectively) or headaches (20.6% and 31.7%, respectively).  Of the 74 participants with ADHD as a main or secondary diagnosis, 32.4% had ongoing stimulants as treatments and they reported a lower prevalence of CWP than those not treated with stimulants (16.7% and 42% respectively) |

**Supplementary Table 5.** Overview of treatment approaches and outcomes in people with mental illness

| **First author + year** | **Setting** | **Study Design** | **Sample Size**  **[N studies**  **N participants]** | **Chronic Pain type/location** | **Chronic pain assessment method** | **Mental illness diagnostic criteria** | **Treatment approaches** | **Treatment outcomes** |
| --- | --- | --- | --- | --- | --- | --- | --- | --- |
| **Anxiety and stress disorders** | | | | | | | | |
| Rometsch-Ogioun El Sount 2019 | Multiple:  psychiatric trauma clinics +  medical  /psychiatric treatment centers +  rehabilitation centers +  community settings | SR & MA | K =15  n PTSD = 10,931 vs n controls = 11,135 | Various chronic pain sites:  headaches, back pain, pain in arms and legs, pelvic pain, stomach pain, joint pain, chest pain | Various, validated scales, clinical assessments | DSM / ICD | One RCT - 6 sessions of Emotional Freedom Technique versus | Pain scores reduced baseline 4.78 (0.44)/10 to 2.94 (0.50)/ 10 after 6 months (p<0.001) |
| **Depression** | | | | | | | | |
| Patel 2024 | Primary care settings | SR & MA | K = 7  n = 891 | Multiple | Neuropathic or inflammatory pain, formally diagnosis without specifying diagnostic methods | Formal diagnosis without specifying diagnostic methods | 7 multicomponent RCTs  3 online psychosocial CBT: 2 tested similar version of the ‘Pain Course’ intervention, and 1 ‘Get Back’, Collaborative care  1 collaborative care, 1 integrated medical group visits, 1optimized pharmacological and psychological pain management and 1 psychoeducational intervention | Nonsignificant effect on chronic pain at post intervention (SMD=0.27, 95% CI -0.08-0.61) and follow-up (SMD=0.13, 95% CI -0.3-0.56) |
| You 2021 | Not specified | SR & MA | K = 8  n = 636 | Not specified | Not specified | Not specified but diagnoses in included studies established via clinical diagnosis | 7 used manual acupuncture either alone or combined with conventional drug therapy (duration ranged from 30 days/4 weeks to 2 months) and 1 used electroacupuncture for 6 weeks | Single acupuncture treatment and drug have the same effect in improving depression (MD=-0.14, 95% CI -0.88-0.59) and pain (MD=-0.42, 95% CI-1.10, -0.27)  Acupuncture combined with drugs is more effective than single-drug treatment in improving depression (MD=-2.95, 95% -3.55, -2.36) and pain (MD=-1.06, 95% CI -1.65, -0.47) |
| **Dementia** | | | | | | | | |
| Martorella 2023 | Community | RCT | n = 40  n=20 in active tDCS  N=20 in sham tDCS | Not specified | Caregiver-reported chronic pain in the past 3 months and scored ≥3 out of 10 | Early stage ADRD via Clinical Dementia Rating score = 0.5-1.0, MMSE score = 16-23), or blinded/telephone version of MoCA score = 16-26. | Transcranial direct current stimulation | Significantly reduced pain intensity: d=0.69 on NRS; d=1.12 on MOBIDS |
| **Severe mental illness** | | | | | | | | |
| **Mental illness** | | | | | | | | |
| Ma 2024 | Multiple | SR | K = 26  n =2,311 | Multiple | Self-reported or clinically diagnosed pain that lasts for at least 3 months | DSM or ICD or other validated clinical diagnostic methods | **Depression**: 1 body-based intervention, 2 CBT-based intervention, 2 MBI, 1 interpersonal psychotherapy, 2 MCI and 3 pharmacological-based treatment  **PTSD**: 2 body-based intervention, 1 MCI and 1 pharmacological-based treatment  **Comorbid depression and anxiety:** 1 MBI | **Depression:** Of the 11 trials targeted patients with depression, 2/2 CBT-based interventions (small to medium effect), 1/1 body-based intervention (large effect) and 2/3 pharmacological-based intervention reported improvements in pain severity. 1/3 pharmacological treatment reported reduced pain interference. 1/2 CBT reported reduced pain-related disability and improved pain-related efficacy (both small effect). 1/2 MBI also reported improved pain catastrophizing (large effect)  **PTSD**: Of the 4 trials targeted patients with PTSD, 1/2 body-based intervention (very large effect) and 1/1 pharmacological therapy reported improved pain severity. 1/1 pharmacological treatment reported reduced pain interference. 1/1 MCI reported reduced pain-related disability  **Comorbid depression and anxiety:** 1 trial targeted comorbid depression and anxiety using MBI and found no significant effect on pain severity, but improvement in pain interference (large effect) |

ADHD = attention deficit hyperactivity disorder; ADRD = Alzheimer's disease and related dementias; ANX = anxiety disorders; BPD = Bipolar Disorders; CBT = cognitive behavioral therapy; CI = confidence interval; DSM = Diagnostic and Statistical Manual of Mental Disorders; ICD = International Classification of Diseases; MA = meta-analysis; MDD = major depressive disorders; MMSE = Mini-Mental Status Exam; MOBIDS= Mobilization-Observation-Behavior-Intensity-Dementia Scale; MoCA = Montreal Cognitive Assessment; NDI = Neck Disability Index; NOS = not otherwise specified; NRS = numeric rating scale; OR = odds ratio; PTSD = posttraumatic stress disorder; SD = standard deviation; SMD = standardized mean difference; SNPs = single nucleotide polymorphisms; SR = systematic review, TAU = treatment as usual; VAS = visual analogue scale

**Supplementary Table 6.** Summary of Treatment Evidence for Chronic Pain Across Mental Disorders

| **Treatment** | **Mental disorder** | **Effect direction** | **Effect size** | **Quality of evidence** |
| --- | --- | --- | --- | --- |
| CBT | Depression | Positive | Small to moderate | High |
| Body-based interventions | PTSD | Positive | Large | High |
|  | Depression | Positive | Large | High |
|  | Comorbid depression and anxiety | Positive | Large | High |
| Pharmacological treatments | PTSD | Positive | ES not reported | High |
|  | Depression | Mixed | Small | High |
|  | ADHD and/or autism | Positive | ES not reported | Moderate |
|  | SMI | Mixed | ES not reported | Moderate |
| Emotional Freedom technique | PTSD | Positive | ES not reported | Moderate |
| tDCS | Dementia | Positive | Moderate to large | High |
| Multimodal interventions | Depression | Positive | Small | High |

ADHD = attention deficit hyperactivity disorder; CBT = cognitive behavioral therapy; ES = effect size; PTSD = posttraumatic stress disorder; tDCS = transcranial direct current stimulation
